# Does trazodone reduce anaesthetic agent requirement in dogs?

**DOI:** 10.18849/ve.v7i3.530

**Published:** 2022-08-03

**Authors:** Gemma Stewart+

**Keywords:** ANAESTHESIA, ANAESTHETIC INDUCTION AGENT, CANINE, MINIMUM ALVEOLAR CONCENTRATION, TRAZODONE, VOLATILE AGENT

## Abstract

PICO question

In dogs undergoing anaesthesia, does the use of oral trazodone given 2 hours before induction of anaesthesia reduce injectable or inhalant anaesthetic agent requirements?

Clinical bottom line

Category of research question

Treatment

The number and type of study designs reviewed

Two randomised controlled trials were critically appraised

Strength of evidence

Moderate

Outcomes reported

Trazodone was shown to have a significant isoflurane minimum alveolar concentration (MAC) sparing effect for isoflurane. There is also evidence to suggest trazodone has a similar effect on the cardiovascular system as acepromazine

Conclusion

Trazodone should be considered as part of a multimodal approach to anaesthesia in dogs to reduce the injectable and inhalant anaesthetic agent requirements

How to apply this evidence in practice

The application of evidence into practice should take into account multiple factors, not limited to: individual clinical expertise, patient’s circumstances and owners’ values, country, location or clinic where you work, the individual case in front of you, the availability of therapies and resources.

Knowledge Summaries are a resource to help reinforce or inform decision making. They do not override the responsibility or judgement of the practitioner to do what is best for the animal in their care.

## Clinical scenario

Patients undergoing anaesthesia require handling, restraint and often painful procedures ahead of anaesthesia in veterinary practice, and many patients display signs of stress and anxiety in response. Trazodone is a recognised anxiolytic (Gilbert-Gregory et al., 2016) that is frequently being used preoperatively in dogs. The purpose of this Knowledge Summary is to analyse the evidence behind the potential benefits of the use of trazodone ahead of general anaesthesia. Trazodone is not licensed for use in dogs in the UK, therefore great consideration must be taken ahead of its administration as the Veterinary Medicines Directorate (GOV.UK., 2022) cascade must be followed when using trazodone.

## The evidence

Two relevant studies were found that addressed the PICO question. One study on research dogs comparing the difference between the administration of trazodone and no premedication on minimum alveolar concentration (MAC) (Hoffman et al., 2018) and one based in clinical practice comparing trazodone to acepromazine as a premedicant for orthopaedic surgery (Murphy et al., 2017). There is evidence that the use of trazodone provides a small reduction in volatile anaesthetic agent requirement but a lack of evidence to support any reduction in injectable induction agent requirements. There is a requirement for further research to directly study trazodone in a wider population of dog breeds and ages with fewer confounding factors and larger study sizes.

## Summary of the evidence

**Hoffman et al. (2018):** 

Population:	Adult healthy hound dogs <1 yrs old, 50% male 50% female.
Sample size:	Six dogs.
Intervention details:	Randomised into two blinded observer groups: 8 mg/kg trazodone administered orally 2 hours prior to induction of anaesthesia (n = 3). No premedication (n = 3). Anaesthesia was induced with 6 mg/kg propofol as an intravenous bolus, the trachea was intubated with a cuffed endotracheal tube and anaesthesia was maintained with isoflurane in oxygen >95%. Volume controlled ventilation was used at a rate of 10 breaths per minute, to achieve a tidal volume of 10–15 ml/kg to maintain normocapnia (PE’CO_2_ 30–45 mmHg). The same monitoring and active heating devices were used for each participant to maintain body temperature between 37.0–39.0°C. Invasive blood pressure monitoring was used throughout the procedure. There was a 60 minute calibration period to allow the elimination of the induction agent propofol prior to minimum alveolar concentration (MAC) determination. MAC was determined using an iterative bracketing technique where repeated electrical stimulation was administered via two 24 gauge 10 mm insulated needle electrodes, inserted 2 cm apart in the buccal mucosa at 50 V 5 Hz over a minute. If gross purposeful movement was made (objective assessment of gross purposeful movement, defined as lifting of the head and / or repeated limb movements, by a single observer), stimulation was ceased immediately. If gross purposeful movement was detected, the concentration of isoflurane was increased until the end expired isoflurane concentration (F_E_Iso) was 10% higher, the animal was then given 15 minutes to equilibrate before proceeding to repeat the stimulus. If gross purposeful movement was not detected, isoflurane concentration was reduced until a 20% decrease in F_E_Iso was achieved, the animal was then given 15 minutes to equilibrate before proceeding to repeat the stimulus. MAC was recorded as the mean F_E_Iso between when gross purposeful movement occurred and when it did not in response to electrical stimulation. MAC was determined twice per anaesthetic and a third time if the results were not within 10% of each other. There was a 7 day washout period between protocols.
Study design:	Randomised controlled trial, crossover design.
Outcome studied:	MAC of isoflurane in dogs. Haemodynamic variables of dogs following the administration of trazodone.
Main Findings (relevant to PICO question)	MAC of isoflurane in dogs. Haemodynamic variables of dogs following the administration of trazodone.
Limitations:	Low sample size of the same breed, all under a year in age, may alter the MAC of isoflurane. Large variation in results. No measurement of trazodone plasma concentration, oral bioavailability may have varied. Baseline MAC of isoflurane reported in this study is lower than others (Barletta et al., 2016). MAC of isoflurane is dependent on the frequency of the electrical stimulation, whilst identical to that used in other studies, may not be representative of MAC with a greater noxious stimuli such as in invasive surgical procedures. The frequency of the stimulus used may not have been supramaximal leading to animals being at too light a plane of anaesthesia and therefore more close to an approximation of MAC awake.

**Murphy et al. (2017):** 

Population:	Systemically healthy, client-owned dogs presenting for cruciate surgery. Exclusion criteria were a seizure history or treatment with other medications within five times the half-life of a given product.
Sample size:	30 dogs.
Intervention details:	Participants randomised into two groups: Acepromazine 0.01–0.03 mg/kg intramuscularly 30 minutes before induction of anaesthesia (n = 15). Trazodone 5 mg/kg if >10 kg or 7 mg/kg if ≤10 kg orally 2 hours prior to induction of anaesthesia (to the nearest quarter of a 50 mg tablet) (n = 15). Both groups received: 1 mg/kg morphine sulphate intramuscularly 30 minutes prior to induction. 4–6 mg/kg propofol was administered to effect, intravenously, over 20–30 seconds, to induce anaesthesia. Jaw tone, palpebral reflex and reaction to the laryngoscope touching the tongue were assessed by an independent clinician who was not aware that propofol was being studied, until a sufficient plane of anaesthesia was achieved to enable orotracheal intubation. Participants had their heart and respiratory rates, mucous membrane colour and capillary refill time, pulse oximetry, end tidal side stream capnography, electrocardiogram (ECG) output, temperature anaesthetic depth and oscillometric non-invasive blood pressure monitored throughout the procedure. When in the operating theatre, participants had an arterial catheter placed to enable direct blood pressure monitoring. Participants underwent either a tibial plateau levelling osteotomy (TPLO) (n=22) or a tibial tuberosity advancement (TTA) (n=8) including pre and postoperative radiography and a preoperative epidural of 0.1 mg/kg preservative free morphine and 0.5 mg/kg bupivacaine. Anaesthesia was maintained using isoflurane or sevoflurane, manual ventilation was commenced if indicated based on capnography. Blood pressure was initially monitored at 5 minute intervals using a non-invasive oscillometric device until an arterial catheter could be called following induction to enable direct arterial blood pressure monitoring throughout the procedure. Intraoperative hypotension was managed with fluid boluses or medication. Adjustments were made in the delivery of inhalant in response to hypotension and / or inadequate depth of anaesthesia. Concurrent bradycardia and hypotension were treated with glycopyrrolate 0.005 mg/kg. Additional analgesia was provided with nitrous oxide or constant rate infusion administration if required.
Study design:	Randomised controlled trial.
Outcome Studied:	Volume of propofol required to induce anaesthesia was recorded by an observer. Haemodynamic variables.
Main Findings (relevant to PICO question):	No significant difference in propofol dose for induction of anaesthesia between groups (p = 0.33). No significant difference in isoflurane vapouriser setting during anaesthesia between groups (p = 0.50). No significant difference in cardiovascular variables between groups for dogs that received inotropic agents heart rate (p = 0.78), mean arterial pressure (p = 0.60), systolic arterial pressure (p = 0.24) or diastolic arterial pressure (p = 0.96).
Limitations:	Varying degrees of disease requiring variation in procedure performed and pain can cause differences in anaesthetic agent requirements. Dose of acepromazine was selected based on anaesthetist’s preference and demeanour of dog, those receiving higher doses may have required less induction or inhalant anaesthetic agents. Trazodone plasma levels were not measured and oral bioavailability may have varied. Intraoperative medications varied between participants, affecting the inhalant anaesthetic agent being administered and therefore cardiovascular parameters. Morphine dose administered as premedication may have caused enough sedation and propofol sparing to make the differences between the two groups negligible. Rapid induction may have resulted in a greater volume of propofol being administered than actually required. Drugs and procedures were performed by multiple members of staff at varying levels of qualification including students under supervision causing variations in technique and efficacy of treatments. Fraction of expired inhalation agent was not measured in all participants so results are based on a vapouriser setting that may not accurately represent what was delivered to the participant. Use of different inhalation anaesthetic agents reduced the number of participants analysed. Varying success of epidural anaesthesia adjunct, the use of a consistent highly trained person may have reduced the variation but ultimately when designing the study a more standardised or less invasive procedure may have provided more reliable results.

## Appraisal, application and reflection

The randomised controlled trials appraised are designed appropriately to address the PICO question (EBVM Network, 2021). Hoffman et al. (2018) used no treatment as a comparator and Murphy et al. (2017) used a recognised premedication acepromazine as a comparator. The level of evidence appraised is level two based on the Oxford Centre for Evidence-based Medicine Levels of Evidence (CEBM, 2009) which provides fairly robust evidence towards answering the PICO question. There is no higher level of evidence published that was suitable to answer the PICO question. Both studies are based on objective assessments. Randomly allocating the treatment group reduces potential bias from allocation and with the observer.

The evidence appraised demonstrates trazodone provides an isoflurane minimum alveolar concentration (MAC) sparing effect in anaesthetised dogs (Hoffman et al., 2018) and has similar cardiovascular effects to acepromazine in anaesthetised, healthy dogs (Murphy et al., 2017). Hoffman et al. (2018) provides supporting evidence that trazodone does reduce the MAC of isoflurane reported as 1.02 ± 0.11% reducing to 0.85 ± 0.17% with the addition of trazodone to the protocol (p = 0.01, 95% confidence interval -0.25–^-^0.05). Murphy et al. (2017) could find no significant differences between trazodone and acepromazine, a strong comparator which has been shown independently to have a MAC sparing effect (Monteiro et al., 2016). However, there were multiple confounding factors which may have influenced the results. Primarily, this study reported on the reduction in propofol dose required for induction of anaesthesia. No difference was found between the acepromazine and trazodone groups of patients. Acepromazine has been shown to reduce the propofol dose required for the induction of anaesthesia (Dantino et al., 2021), therefore trazodone may have a similar effect, although difficult to determine due to confounding factors of varied acepromazine doses and rate of propofol administration. Acepromazine is known to have a reducing effect on blood pressure in dogs (Grasso et al., 2015), therefore by having no significant difference in cardiovascular variables, trazodone may also have an impact on blood pressure. Results demonstrated by Hoffman et al. (2018) show that blood pressure and heart rate were similar in cases that had received trazodone or higher levels of isoflurane, which would suggest trazodone could have cardiovascular effects.

Both papers accounted for all of the animals included in the trial in the conclusion. Hoffman et al. (2018) report results from all of the animals included in the trial. Murphy et al. (2017) state some of the participants in their trial were excluded from the results, mostly because a standardised protocol was not used throughout the procedure, for example the use of isoflurane (n = 28) versus sevoflurane (n = 2). Hoffman et al. (2018) used a crossover design to the study, enabling a direct comparison between interventions in all participants. Other than the intervention and the comparator, the groups were treated equally in both studies appraised.

There are limitations to the research available. Inclusion and exclusion factors are well defined in both papers, but participants are not fully representative of all patients undergoing the multitude of procedures performed in practice. Whilst they are of the correct species, the evidence focuses solely on systemically healthy dogs. Potential candidates with concurrent disease were excluded from both studies, limiting the application of the research in clinical practice where many of the patients suffer with concurrent disease, highlighting an area for further research. There are weaknesses in the Murphy et al. (2017) study design, despite being a randomised control trial, that limit the conclusions drawn from the research. No control was used for comparison meaning it cannot be determined whether or not trazodone reduced the quantity of propofol required for the induction of anaesthesia. The comparator, acepromazine, had a 200% variation in dose at the discretion of the anaesthetist, affecting the validity of the results obtained by comparison.

Further research would be desirable to improve the external validity of the evidence presented. The multiple variables that could be influencing the results in the Murphy et al. (2017) study’s conclusion that there are no significant differences between acepromazine and trazodone are concerning and affect the level of reliability of the results. Hoffman et al. (2018) do provide reliable results although on a small scale with a narrower population demographic.

All of the evidence appraised is based on a single dose of orally administered trazodone 2 hours before the induction of anaesthesia, administered in the clinical setting. As an anxiolytic drug, if administered ahead of the stressor that is travelling and visiting a veterinary practice the effects may be improved and provide a more practical approach to the administration of trazodone as part of a pre-anaesthetic protocol. Hoffman et al. (2018) used an 8 mg/kg dose 2 hours before the induction of anaesthesia and Murphy et al. (2017) used a 5–7 mg/kg dose depending on body weight. The BSAVA formulary stated dose for use in dogs to treat chronic anxiety is ‘5–10 kg, 25 mg p.o. q24h; 11–20 kg, 50 mg p.o. q24h; >21 kg, 100 mg p.o. q24h’ (Ramsey, 2017). Jay et al. (2013) report the time to maximum plasma concentration following oral administration as 445 minutes ± 271 minutes which would mean the 2 hours allowed prior to induction would be inadequate. This could also be problematic when considering adding trazodone as part of premedication as to reach maximum plasma concentration, it would need to be administered 7.5 hours before induction.

Beneficial effects of the treatment were identified and no significant adverse effects were noted with the administration of trazodone when compared with no intervention or acepromazine. Murphy et al. (2017) reported one dog experienced priaprism 24 hours post administration of trazodone which was resolved with treatment, this is a rare side effect also noted in humans (Abber et al., 1987). Trazodone has been shown to reduce stress in postoperative patients (Gruen et al., 2014). This could be extrapolated to patients confined ahead of anaesthesia, who may therefore require less anaesthetic agents to induce and maintain a suitable depth of anaesthesia. There could be an argument for combining it as part of a multimodal approach to the pre-anaesthetic protocol, however, the reported MAC reduction of trazodone is less than other agents such as alpha-2-agonists (Sinclair, 2003) or opioids (Credie et al., 2010).

In conclusion, there is some strong evidence to support the intervention of trazodone to reduce the dose of inhalant anaesthetic agent administered. Consideration must be taken regarding the use of the cascade due to lack of licensing and the potential for only a modest reduction in MAC. Administration orally 2 hours before the induction of anaesthesia, may be an inadequate time to reach maximum effect. Further research would be beneficial to establish a stronger argument for the inclusion of trazodone in a multimodal anaesthetic protocol.

## Methodology Section

**Search Strategy:** 

Databases searched and dates covered:	CAB Abstracts on OVID Platform 1973–December Week 49 2021Medline on OVID Platform 1946–December Week 49 2021PubMed 1900–December 2021Web of Science 1970–2021VetMed Resource 1972–2021
Search strategy:	CAB Abstracts: (cani* or dog*) (anaesthe* or anesthe* or surgery or volatile agent) (trazodone) 1 and 2 and 3 Medline: (cani* or dog*) (anaesthe* or anesthe*or surgery or volatile agent) (trazodone) 1 and 2 and 3 PubMed:((cani* or dog*) AND (anesthe* or anaesthe* or surgery or volatile agent)) AND (trazodone) Web of Science:TOPIC: (cani* or dog*) AND TOPIC: (anaesthe* or anesthe* or surgery or volatile agent) AND TOPIC: (trazodone) VetMed Resource:(cani* or dog*) AND (anaesthe* or anesthe* or surgery or volatile agent) AND (trazodone)
Dates searches performed:	12 Dec 2021

**Exclusion / Inclusion Criteria:** 

Exclusion:	Articles not relevant to PICO based on title and abstract, single case reports, duplicate articles and author responses.
Inclusion:	Articles written in English which were relevant to the PICO based on title and abstract involving multiple animals.

**Search Outcome:** 

Database	Number of results	Excluded – Not relevant to PICO	Excluded – Single case report	Excluded – Duplicate article	Excluded – Author response	Total relevant papers
CAB Abstracts	10	6	1	1	0	2
Medline	7	5	0	2	0	0
PubMed	12	9	0	2	0	0
Web of Science	10	8	0	2	0	0
VetMed Resource	10	6	1	3	0	0
Total relevant papers when duplicates removed						2

## Conflict of interest

The authors declare no conflicts of interest.
